# Checkpoint Inhibitors in Cancer Therapy: Clinical Benefits for Head and Neck Cancers

**DOI:** 10.3390/cancers14204985

**Published:** 2022-10-11

**Authors:** Tobias Ettl, Matthias Grube, Daniela Schulz, Richard Josef Bauer

**Affiliations:** 1 Department of Oral and Maxillofacial Surgery, University Hospital Regensburg, 93053 Regensburg, Germany; 2 Department of Hematology and Oncology, University Hospital Regensburg, 93053 Regensburg, Germany; 3 Center for Medical Biotechnology, Department of Oral and Maxillofacial Surgery, University Hospital Regensburg, 93053 Regensburg, Germany

**Keywords:** PD-L1, immune checkpoint, clinical trial, head and neck, combination therapy, ICI, head and neck cancer

## Abstract

**Simple Summary:**

In recent years, checkpoint inhibitor treatment of tumors has caused a stir. The response of patients with metastases showed outstanding success for some cancer types. However, many tumor types develop resistance strategies to evade this therapeutic application. This review provides an overview of the potential and broader treatment options that have emerged in recent years with immune checkpoint inhibition (ICI) treatment. Here, a major focus is placed on treatment with ICIs and combination therapies for head and neck tumors. This review offers a comprehensive overview of clinical trials for ICI (combination) therapies in advanced stages, as well as clinical trials in their early stages for head and neck tumors.

**Abstract:**

Recently, considerable progress has been achieved in cancer immunotherapy. Targeted immune checkpoint therapies have been established for several forms of cancers, which resulted in a tremendous positive impact on patient survival, even in more advanced tumor stages. With a better understanding of cellular responses to immune checkpoint therapies, it will soon be feasible to find targeted compounds which will make personalized medicine practicable. This is a great opportunity, but it also sets tremendous challenges on both the scientific and clinical aspects. Head and neck tumors evade immune surveillance through various mechanisms. They contain fewer lymphocytes (natural killer cells) than normal tissue with an accumulation of immunosuppressive regulatory T cells. Standard therapies for HNSCC, such as surgery, radiation, and chemotherapy, are becoming more advantageous by targeting immune checkpoints and employing combination therapies. The purpose of this review is to provide an overview of the expanded therapeutic options, particularly the combination of immune checkpoint inhibition with various conventional and novel therapeutics for head and neck tumor patients.

## 1. Introduction

Expanding the range of treatment options for head and neck squamous cell carcinoma (HNSCC) is of considerable significance, particularly for advanced or refractory tumors. After all, this tumor type is the ninth most common cancer worldwide. In the United States, there are approximately 53,000 new cases of HNSCC and more than 10,000 deaths annually [1]. 

In the last thirty years, besides noxious-induced HNSCC, human papillomavirus (HPV) and Epstein-Barr virus (EBV) gained increasing importance for the development of squamous cell carcinoma of the oropharynx and nasopharynx, respectively [2,3,4]. Furthermore, HNSCC can affect all areas of the squamous mucosa starting from the lips through the oral cavity, nasal cavity, paranasal sinuses, nasopharynx, oropharynx, hypopharynx, and larynx [5]. Most HNSCC patients have locally advanced disease with a high risk of recurrence, and regional and distant metastases occur in approximately 30% and 10% of patients, respectively [6]. The tumor type itself is a biologically diverse and genomically heterogeneous disease, and therefore the treatment strategies are multimodal. The surgical resection of primary tumor and draining lymph nodes, followed by risk-adapted adjuvant radiation with or without platinum-based chemotherapy, is still the standard treatment for locally advanced but resectable tumors, while definitive concurrent chemoradiation remains the main treatment modality for advanced, non-resectable, or metastasized HNSCC. Both surgery and chemoradiation often drastically impact the patients’ quality of life in terms of functionality and aesthetics [7]. 

In locally recurrent and/or metastatic (R/M) disease, platinum-based dual chemotherapy combined with the anti-epidermal growth factor receptor (EGFR) monoclonal antibody cetuximab was the standard of care (SOC) in the first-line setting for over a decade. In the EXTREME trial (NCT00122460), the additional administration of cetuximab prolonged median progression-free survival (PFS) from 3.3 to 5.6 months (HR 0.54; *p* < 0.001), median overall survival (OS) from 7.4 to 10.1 months (HR 0.80; *p* = 0.04), and response rates from 20% to 36% (*p* < 0.001) [8]. Until recently, second-line therapies available were cetuximab, methotrexate, and a taxane, each associated with response rates of 10–13% and a median PFS of 2–3 months, with no clear evidence of OS improvement [9]. The five-year survival for HNSCC patients (except EBV-related nasopharyngeal) is still only 40–50% for noxious (smoking and alcohol) HPV-negative tumors at all stages. The median overall survival (OS) for patients with recurrent/metastatic (R/M) disease is 10–13 months [6].

## 2. Drug Resistance

Drug resistance remains one of the most important factors preventing the cure of cancer patients. While many chemotherapeutic agents have shown and continue to show frequent success, tumors go into remission relatively quickly, but rapidly develop strategies to survive, become resistant, and relapse. To address the challenge of single-agent resistance, combinations of agents with overlapping mechanisms of action were employed. This approach worked excellently in breast cancer, lymphoma, and testicular cancer, and has been continuously developed [10,11,12]. The therapies steadily improved the prevention of early tumor recurrence. This was due, in part, to the administration of chemotherapies at shorter intervals, higher doses of therapy, along with growth factors to accelerate recovery from chemotherapy induced myelosuppression [13,14]. These strategies were successful, but they were not enough to provide a complete remission for many tumor types. 

A further step to improve anti-tumor treatment was the invention of targeted therapies, which block tumor cell growth by interfering specifically with molecules inside or on the surface of tumor cells which are important for the regulation of cancer cell growth, division, and spreading [15]. The main types of targeted therapy are small-molecule drugs and monoclonal antibodies (mAb). 

More recently, significant progress has been made by targeting the negative regulators (checkpoints) of the adaptive immune system, as well as utilizing mAbs (such as anti-CTLA-4 and anti-PD-1/PD-L1). With the help of these therapies, there have even been cures of various tumor entities [16]. 

However, similar to conventional chemotherapy, resistance develops under the influence of these targeted immunological therapies. For this reason, various approaches exist to overcome the development of drug resistance with monotherapy. Combination with various other therapeutic strategies seems to offer a good solution here. Therefore, multiple clinical trials are currently underway, combining therapies of immune checkpoint inhibitors with conventional chemo- or radiotherapy or/and with the targeted elimination of signaling pathway key molecules. As a result, combination therapies should at best add to and increase the tissue tumor mutation burden (tTMB) and microsatellite instability, and subsequently induce neo-antigen expressions in order to trigger a T-cell-mediated immune response. This aims to convert HNSCCs, generally known as “cold tumors” with low local immune response, into “hot tumors” with a higher local immune reaction. Independently, clinical studies have demonstrated higher tTMB to be associated with higher numbers of neoantigens. This was in some solid cancers associated with an increased response to immune checkpoint therapy [17]. New findings in clinical studies reveal tTMB as biomarkers when treated with pembrolizumab, as further discussed in Section 5 [18].

**Figure 1:**
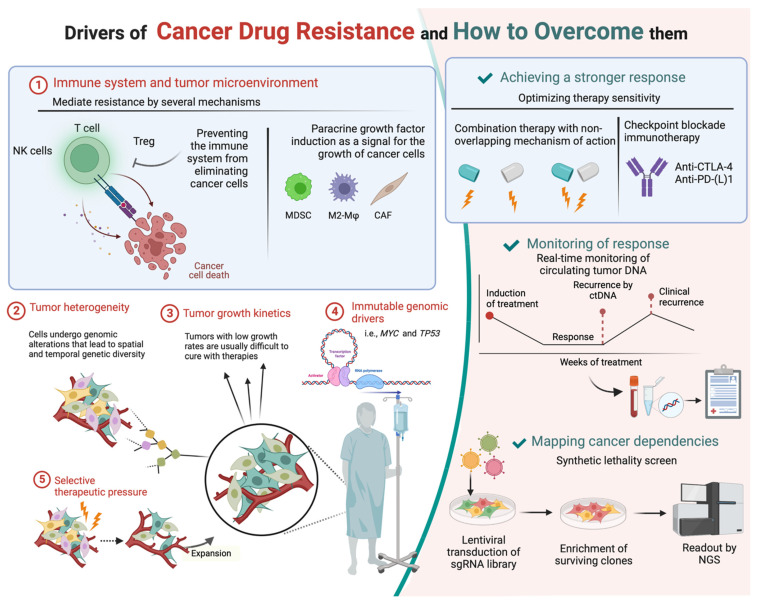
Tumor resistance to therapeutics is a big issue that often prevents a complete cure for different tumor types. This diagram shows exemplary strategies of tumors to develop resistance and strategies of clinical therapy to suppress or eliminate resistance by new therapeutic developments and approaches. In this review, we focus on therapeutic combination strategies in head and neck cancer that are currently being investigated in clinical trials to circumvent tumor immunological resistance development (image created with BioRender.com).

The review presented here provides a comprehensive overview of the various therapeutical approaches. Figure 1 shows examples of some of the important factors that contribute to the development of resistance to tumor therapies. It also illustrates ways to counteract this resistance. There are currently countless clinical trials underway investigating the efficacy of immune checkpoint inhibitors in combination with individualized chemotherapy and radiotherapy. In addition, very promising therapeutic tools, such as the real-time monitoring of circulating tumor DNA and synthetic lethality screens, have emerged in recent years.

## 3. HNSCC Tumor Microenvironment (TME)

In recent decades, it has become increasingly clear that non-cancerous cells surrounding the tumor and the extracellular matrix (ECM) proteins, which together form the TME, play a critical role in tumorigenesis, the progression of aggressive tumors, and the development of treatment resistance [19,20]. The TME is enriched with immunomodulators, in addition to various nutrients, chemokines, cytokines, growth factors, intermediate metabolites, hormones, and growth factors. These factors are secreted by the tumor itself as well as the surrounding stroma. Thus, the TME provides a beneficial setting for the progression of the tumor and the emergence of resistance [21,22]. Newly established treatment approaches targeting both the TME and cancer cells have led to greater treatment efficacy and a better prognosis for patients [23].

Intact immune surveillance is needed to control cancer development. For tumor cells to spread, they must hide from the immune system and escape its surveillance. Through local cellular selection, many solid and non-solid tumor entities develop sophisticated mechanisms to prevent the local and systemic activation of the immune system. 

HNSCC is a tumor type associated with strong immune infiltration [24], with T cells not being excluded from the microenvironment in contrast to many other tumor types. Immune responses are known to be suppressed by the presence of Tregs. Thus, they are also part of the immune evasion mechanism in head and neck tumors [25]. Consequently, HNSCC tumors have to develop a suppressive milieu. One strategy to achieve this is to upregulate immunosuppressive cytokines, such as transforming growth factor beta (TGF-β), interleukin (IL)-6, or IL-10 [26]. In addition, STAT3 and NFκB pathway activation is often downregulated [27]. At the same time, HNSCC tumors frequently express aberrant HLA class I antigens, resulting in T cell tolerance [28].

With our current state of knowledge, we understand the critical role that TME plays for the majority of solid tumors during advanced disease development much better [29]. The TME of HNSCC is of major significance for tumor progression and needs to be addressed regarding clinical and therapeutic strategies.

The alteration or defective processing of non-cellular components (such as collagen type I; fibronectin; laminin; tenascin; and non-physiological conditions of pH, oxygen, and interstitial pressure) represents a basic building block towards aberrant cell signaling and resulting tumor progression. The chemotactic attraction of diverse cell types, such as bone marrow stem cells, adipose stem cells, cancer-associated fibroblasts (CAFs), endothelial cells (ECs), adipocytes, neuroendocrine cells, and hematopoietic and lymphatic cells [30,31], further establishes a favorable environment for tumor growth, metastasis, and the development of resistance to therapy [22].

The relationship between tumor cells and stromal cells Is based on mutual interaction. Tumor cells, on the one hand, attract stromal cells, and the stromal cells, in turn, supply the tumor cells with nutrients, hormones, cytokines/chemokines, intermediate metabolites, and growth factors, thus promoting their invasion, migration, metastasis, proliferation, and survival [24,32,33,34,35,36,37,38,39].

Ultimately, with this type of assemblage, various components of the immune system, such as complement factors, lymphocytes (TILs), and their subsets (including CD8+ cytotoxic T cells, CD4+ helper T cells, CD163+ and CD68+ macrophages and MDSCs, CD57+ NK cells, and FOXP3+ T regulatory cells (Tregs) [40,41,42,43]) often fail to eliminate the tumor, but rather contribute to the stabilization of tumor progression and help to evade immune recognition [36,44]. All currently available data indicate that although the TME contains immunostimulatory components at premalignant states, it provides the foundation for an immunosuppressive environment for advanced tumor stages [45,46,47].

## 4. Development towards ICI Combination Therapy

In recent years, immunotherapy has opened new treatment options for HNSCC. Immune therapy should increase the activity of the immune system to destroy cancer cells [48]. Immune checkpoint inhibitors (ICIs) make up a widely effective class of immunotherapies that block inhibitory immune checkpoint signaling pathways to reactivate immune responses against cancer. Since 2016, two immunotherapeutic agents have been approved by the U.S. Food and Drug Administration (FDA) for patients with refractory R/M HNSCC who do not respond to platinum-based therapy. Both nivolumab (Opdivo, Bristol-Meyers Squibb) and pembrolizumab (Keytruda, Merck) are monoclonal antibodies used against the programmed cell death-1 (PD-1) receptors. The binding of the PD-1 protein, mostly expressed by T cells, to PD-L1, which is frequently expressed by tumor cells, results in the suppression of T cell immunologic responses and serves as a mechanism to bypass the tumor immune system [49,50]. Anti-PD-1/PD-L1 ICIs can block suppressive signaling through the PD-1/PD-L1 pathway and enhance tumor immune activity [28,51]. 

The European Commission subsequently approved nivolumab in 2017 for the treatment of the same patient population, followed by the approval of pembrolizumab as a type of monotherapy soon after for the treatment of recurrent or metastatic HNSCC in adults whose tumors express PD-L1 with a tumor proportion score of ≥50% and who have progressed on or after platinum-containing chemotherapy.

In the following table (Table 1), we list currently ongoing advanced-stage clinical trials (phase III) involving combination therapy with ICIs.

## 5. Finding Biomarkers

The best therapies are of no use if they have no effect on the patient or are associated with undesirable side effects. Therefore, the identification of biomarkers is crucial to target the patients who will benefit and respond most to a given regimen.

PD-L1 expression has shown to be a potential biomarker. Patients with PD-L1 expression > 1% treated with nivolumab presented a hazard ratio for death (HR) of 0.55 (95% CI 0.36–0.83) compared to standard therapy, whereas the HR in patients with PD-L1 expression < 1% was 0.89 (95% CI 0.54–1.45) [60]. In the KEYNOTE-048 study, the effectiveness of pembrolizumab treatment was dependent upon the PD-L1 combined positive score (CPS). This score combines tumoral together with the PD-L1 expression of immune cells [52]. At present, PD-L1 expression represents the sole biomarker employed in routine clinical practice.

The KEYNOTE-012 trial reported superior outcomes in HPV-positive patients compared to HPV-negative ones. This study revealed an ORR of 24% (95% CI, 13–40%) among patients who were found to have HPV-associated disease. In contrast, patients who did not suffer from HPV-associated disease showed an ORR of only 16% (95% CI, 10–23) [61].

Tissue tumor mutation burden (tTMB) refers to the number of somatic mutations per megabase of genome sequence examined and varied across cases. For patients with solid cancers treated with pembrolizumab, KEYNOTE-158 showed objective responses in 29% of patients with high tTMB (>10 mut/Mb, 95% CI 21–39) compared to 6% in the non-TMB-high group (CI 95% CI 5–8) [18].

In HNSCC, high TMB levels were correlated to higher response rates and longer overall survival after immunotherapy, particularly in HPV-negative tumors, whereas this association was not significant in HPV-positive tumors. Among responders to anti–PD-1/PD-L1 treatment, the most common mutations were in *SMARCA4*, *TP53*, *KMT2D*, and *NOTCH1* genes [18,62,63].

Somatic mutation load (ML) and interferon gamma (IFN-γ) gene expression profile also proved to be biomarkers. Regarding the response to pembrolizumab, KEYNOTE-012 in HPV- and EBV-negative patients showed that ML and IFN-γ expression profiles were independent predictively and highly associated with overall survival. Furthermore, the *INF-γ* gene expression pattern also appears to be a predictor in HPV- and EBV-positive patients [64].

## 6. Immune Checkpoint Inhibitors

The activity of T cells is tightly regulated. In this context, the CD28 receptor family plays a major role. Two members, CD28 and ICOS (inducible T cell co-stimulator), act as positive regulators of T cells, and three proteins, BTLA [65], CTLA-4, and PD-1, cause inhibition. PD-1 is a receptor expressed on activated T and B cells, monocytes, and a subset of thymocytes.

Regulatory T cells use the PD-1 signaling axis to control suppression of T cells using PD-L1 and PD-L2 as ligands expressed on their surface. They are expressed on antigen-presenting cells (APCs), epithelial cells, and endothelial cells, as well as on activated lymphocytes. The result of the interaction between PD-1 and PD-L1 is a decrease in cytokine production and the induction of apoptosis in T lymphocytes.

In some tumor entities, the upregulation of PD-L1 can occur under certain circumstances. Cancer cells exploit this mechanism to hide from the host immune system by inactivating T cell function. In recent years, antibodies directed against this signaling axis have been clinically successful in enhancing the T cell response and enabling the immune system to effectively fight intrinsic tumors [66,67]. In HNSCC, PD-L1 expression is reported between approximately 50 and 100%, which is relatively high [68].

### 6.1. CTLA-4 Inhibitors

Ipilimumab, an IgG1 monoclonal antibody (mAb), targets CTLA-4 and was approved in 2011 to treat patients with metastatic melanoma [69]. Clinical trials have shown that monotherapy with ipilimumab (10 mg/kg) can lead to improved OS rates [70] and durable objective response in patients with advanced melanoma [71]. In addition to ipilimumab monotherapy, in a phase 3 trial in patients with advanced melanoma (NCT01844505), this CTLA-4 inhibitor in combination with nivolumab resulted in longer progression-free survival (PFS) and a higher objective response rate (ORR) [72]. The nivolumab–ipilimumab combination resulted in an OS of 58% at 3 years, with the monotherapies each with nivolumab at 52% and ipilimumab at 34% being lower [72].

Ipilimumab is approved in combination with nivolumab (ipi/nivo) for adjuvant treatment in patients with melanoma and as monotherapy or as ipi/nivo for patients with unresectable or metastatic melanoma. Furthermore, ipi/nivo therapy can be used to treat patients with several other tumors, such as hepatocellular carcinoma (HCC) [73], non-small cell lung cancer (NSCLC) [74], unresectable malignant pleural mesothelioma [75], advanced renal cell carcinoma (RCC) [76], unresectable or metastatic esophageal squamous cell carcinoma (ESCC), and metastatic colorectal carcinoma (CRC) with high microsatellite instability (MSI-H) or mismatch repair deficiency (dMMR) [77]. Thus, combination therapy was superior to monotherapy with ipilimumab or nivolumab in terms of efficacy, while a higher rate of immune-related adverse events (irAEs) curtailed its clinical use [72].

Recently, a phase 3 randomized trial (CheckMate 651, NCT02741570) evaluated ipi/nivo versus EXTREME as a first-line therapy for R/M HNSCC. Combined immunotherapy has not shown significant OS improvement compared to EXTREME; however, there was some evidence of clinical activity in patients with CPS greater or equal to 1 or 20, as shown by prolonged OS (median and 2 year rates) and durable responses [78].

### 6.2. PD-1 Inhibitors

Nivolumab, pembrolizumab, as well as cemiplimab are fully human IgG4 anti-PD1 mAb.

Recently, for patients with platinum-resistant R/M HNSCC, nivolumab monotherapy was randomized and compared with second-line single agents (docetaxel, methotrexate, or cetuximab) in the phase III trial CheckMate 141. In a population of 361 patients, response rates (RRs) were consistently higher in the nivolumab cohort than in all others, at 13.3%. There were 6 complete responders (CRs) and 26 partial responders (PRs) in the nivolumab group. The median overall survival (OS) was 7.5 months with nivolumab versus 5.1 months in the control group. Patients receiving nivolumab had a statistically significant 30% lower mortality risk (HR 0.70; 95% CI 0.51 to 0.96). Estimated progression-free survival (PFS) improved by nearly 10%, from 9.9% in patients treated by medical decision to 19.7% in nivolumab patients. These data show substantial benefits to patients over a longer period of time, significantly improving quality of life [79]. Contributing to this is the fact that nivolumab has far fewer side effects than standard therapies. Grade 3 or 4 adverse events dropped from 35.1% to 13.1% with nivolumab treatment [60]. These data support the approval of nivolumab as a type of monotherapy for R/M HNSCC patients with disease progression on or after a platinum-based therapy.

Recently, nivolumab treatment beyond RECIST-defined progression (TBP) has been investigated in patients with R/M HNSCC. Of 60 patients suffering from TBP, 15 (25%) had stable disease and 15 (25%) showed a reduction in target lesion size [80]. In conclusion, continuing therapy after disease progression does not pose a safety risk to the patient and the clinical benefit is sustained. This benefit of treatment beyond progression was also evident in other patient groups with advanced melanoma and metastatic renal cell carcinoma [81,82].

Besides the application in patients with HNSCC, monotherapy with nivolumab is also indicative for patients with unresectable or metastatic melanoma, metastatic NSCLC, advanced or metastatic urothelial carcinoma, advanced relapsed or metastatic esophageal squamous cell carcinoma (ESCC), metastatic CRC (MSI-H or dMMR), advanced renal cell carcinoma, and relapsed or refractory (R/R) classical Hodgkin lymphoma (cHL) [83].

Pembrolizumab showed significant anti-tumor activity in HNSCC, resulting in improved ORR with moderate toxicity. It received accelerated approval from the FDA as a type of monotherapy in 2016 for the treatment of R/M HNSCC patients with disease progression on or after platinum-containing chemotherapy after the KEYNOTE-012 phase Ib study showed excellent data, with a response rate of 18% at 9 months (4 CR and 20 PR of 132 HNSCC patients from the expansion cohort), a median OS at 8 months, and a 6-month PFS of 23%. In addition, grade 3 and 4 toxicities occurred in only 9% of patients [84]. A confirmatory phase III study (KEYNOTE-040) compared pembrolizumab to standard therapies in patients with platinum-resistant recurrent/metastatic squamous cell carcinoma of the head and neck. The open-label study was conducted at 97 medical centers in 20 countries. In total, 247 patients were randomly assigned to receive pembrolizumab and 248 were randomly assigned to receive standard therapy (methotrexate, docetaxel, or cetuximab). With pembrolizumab, median overall survival was 8.4 months (95% CI 6.4–9.4), and 6.9 months (5.9–8.0) with standard therapy (hazard ratio 0.80, 0.65–0.98; nominal *p* = 0.0161). Significantly fewer patients again experienced grade 3 treatment-related adverse events, i.e., 13% versus 36% [85].

In 2019, as a result of the phase II KEYNOTE-048 study, the FDA approved pembrolizumab, alone or with chemotherapy, for first-line treatment in patients with unresectable R/M HNSCC. The study assessed pembrolizumab monotherapy or a combination of pembrolizumab with a platin-based agent and 5-fluorouracil in comparison to cetuximab with platin-based and 5-fluorouracil chemotherapy. The pembrolizumab combination proved to be more beneficial for overall survival. Patients survived an average of 13 months compared to only 10.7 months with the cetuximab combination (HR, 0.77; *p* = 0.0034) [52]. To be more specific, the effectiveness of pembrolizumab treatment was dependent upon the PD-L1 combined positive score (CPS). Immune cell PD-L1 expression has shown to be predictive for PD-1 blocking immunotherapy in different types of solid cancer [86]. In the KEYNOTE-048 study, patient survival was highest when the PD-L1 CPS score was greater than 20 (14.9 months for pembrolizumab monotherapy vs. 10.7 for chemotherapy and cetuximab) [52]. Furthermore, when the PD-L1 CPS score was greater than 1, pembrolizumab therapy was superior to cetuximab-based therapy (12.3 months vs. 10.3).

Based on the results of the KEYNOTE-048 study, pembrolizumab as a type of monotherapy or in combination with chemotherapy, was approved as first-line therapy for all patients with R/M HNSCC showing a combined positive score (CPS) ≥1 [52].

In addition to patients with R/M HNSCC, pembrolizumab is used as a type of monotherapy e.g., in patients with metastatic melanoma and NSCLC [87], advanced or metastatic urothelial carcinoma [88], R/R cHL [89], metastatic CRC, ESCC [90], cervical cancer, Merkel cell carcinoma, endometrial carcinoma, cutaneous squamous cell carcinoma (cSCC), and primary mediastinal large B-cell lymphoma.

**Figure 2:**
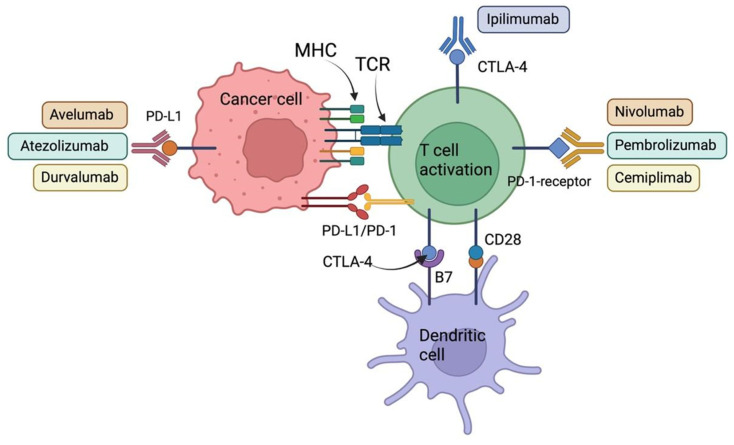
Immune checkpoint inhibitors (ICIs) with FDA approval. The scheme shows pembrolizumab, nivolumab, and cemiplimab (the PD-1 inhibitors); durvalumab, atezolizumab, and avelumab (the PD-L1 inhibitors); and ipilimumab (the CTLA-4 inhibitor). These antibodies are currently the most important ICI treatment options for a number of cancer types (image created with Biorender.com).

Cemiplimab has been approved for the treatment of locally advanced (la) or metastatic (m) cutaneous squamous cell carcinoma (la/m cSCC) [91], basal carcinoma (la/m BCC), and la/m NSCLC. However, its effectiveness in the treatment of patients with R/M HNSCC has not yet been demonstrated [92]. Figure 2 shows an overview of the currently most important approved antibodies in the field of immune chekpoint inhibition.

### 6.3. PD-L1 Inhibitors

Atezolizumab, durvalumab, and avelumab are IgG1 mAbs targeting PD-L1 [93]. Atezolizumab has recently shown clinical efficacy in patients with previously treated, advanced HNSCC. However, further studies are ongoing [94].

A recent phase II study (CheckRad-CD8) demonstrated the feasibility of single-cycle induction treatment with cisplatin–docetaxel and durvalumab combined with tremelimumab (anti-CTLA-4 mAb) and achieved a high biopsy-proven pathologic complete response (pCR) rate in patients with locally advanced HNSCC [95]. Furthermore, a phase II study (HAWK) demonstrated the anti-tumor activity of durvalumab monotherapy in patients with R/M HNSCC. However, two phase III studies (EAGLE and KESTREL) investigating the efficacy of durvalumab or durvalumab combined with tremelimumab (anti-CTLA-4 mAb) versus the standard of care or the EXTREME treatment regimen failed to demonstrate an improvement in the OS of patients with R/M HNSCC (AstraZeneca communication https://www.astrazeneca.com/media-centre/press-releases/2021/update-on-kestrel-phase-iii-trial-for-imfinzi.html (accessed on 21 September 2022) [56]. Recently, avelumab monotherapy has shown clinical efficacy in patients with platinum-refractory/ineligible R/M HNSCC in a phase Ib study (JAVELIN Solid Tumor) [96]. A phase III study (JAVELIN Head and Neck 100) investigated the treatment of avelumab in combination with chemoradiotherapy (CRT) versus placebo combined with CRT in patients with previously untreated, locally advanced, high-risk HNSCC, but the study failed to meet the primary endpoint of PFS improvement [97].

## 7. Checkpoint Regulators and Combination Therapy

### 7.1. The Tumor Microenvironment: Cellular Mechanisms which Inhibit T cell Functions

The basis of ICI treatment primarily relies on T cells. However, T cells can be prevented from performing their immune surveillance correctly or other pathways for immune surveillance evasion can be established in the tumor milieu, which may lead to the failure of ICI therapy.

The evasion of immune surveillance occurs in the tumor milieu in different ways and becomes active at different points. These include an alteration of tumor cell metabolism so that, for instance, indoleamine-2,3-dioxygenase (IDO)-expressing myeloid suppressor cells degrade more tryptophan and produce the immunosuppressive metabolites (e.g., kynurenine), which in turn shuts down T cell expansion [98]. Indoleamine 2,3-dioxygenase 1 (IDO1) was recognized as a catabolizing enzyme that induces T cell-mediated immune tolerance and leads to attenuated immune surveillance. IDO1 has been associated with poor outcomes in the squamous cell carcinoma of the larynx. Moreover, Economopoulou et al. described that IDO1 mRNA expression in circulating tumor cells of HNSCC patients is an independent prognostic factor for clinical outcome [99,100,101].

Tregs, T helper 2 (TH2) cells, and myeloid-derived suppressor cells (MDSCs) in the TME are an additional obstacle which compromises the efficacy of ICI therapies by suppressing CTL and T helper 1 (TH1) cell-mediated tumor immune surveillance [102,103]. The depletion of these cell types has been shown the need to experimentally enhance the anti-tumor immune response and weaken or eliminate resistance to ICI [104].

### 7.2. Strengthening T cell Defense

The WNT-β-catenin signaling pathway in tumor cells also plays a role in the infiltration of tumor infiltrating lymphocytes (TILs) and CD103+ dendritic cells into the TME. In melanoma, it was shown that β-catenin activation could suppress CCL4. This ligand is mainly responsible for the infiltration of immune cells into the TME [105,106].

In addition, it has been observed that a loss of PTEN can promote immune resistance, whereby treatment with a selective PI3Kbeta inhibitor increased the efficacy of anti-PD-1 and anti-CTLA-4 antibodies in murine models [107]. The blockage of immunosuppressive factors, such as IL-10, TGF-β, or VEGF, additionally supports the treatment of ICIs. This activates the migration of dendritic cells and helps to prime T cells.

The expression of the enzyme cyclooxygenase (COX) by tumor cells contributes to the migration of Tregs into the tumor environment and, by creating an inflammatory response mediated by prostaglandin E2, simultaneously promotes immune escape. Therefore, COX-2 inhibition in combination with ICI therapy could also prove promising [108,109].

In addition, tumor glucose and glutamine metabolism were reported to stimulate PD-L1 expression via EGFR and ERK/C-Jun signaling pathways. Consequently, by inhibiting tumor glucose or glutamine metabolism via therapeutic agents, researchers are striving to improve PD-1/PD-L1 antibody therapy and overcome resistance [110].

#### 7.2.1. Costimulatory Agents

The activation of T cells generally requires costimulatory signals. In the absence of these signals, the immune response is weaker, or T cells undergo apoptosis. ICOS, a member of the CD28/B7 superfamily, is a costimulatory signal that promotes T cell expansion, function, and survival. GSK609 is a humanized IgG4 antibody against ICOS which exhibits potent agonist activity and reduces Fc-mediated depletion effects. Consequently, the employment of GSK609 stabilizes ICOS and its costimulatory signal. GSK609 in combination with pembrolizumab showed promising results in HNSCC after platinum failure in a phase I expansion trial [111], but further trials were stopped prematurely.

#### 7.2.2. T Cell Exhaustion

The activation of antigen-presenting cells and CD8+ T cells can occur through eftilagimod alpha. LAG-3 binds to major histocompatibility class II molecules. Eftilagimod alpha in combination with pembrolizumab in second-line therapy resulted in an overall survival of 36% [112]. Once immune checkpoint inhibitor failure occurs in patients with recurrent metastases, an ongoing trial is testing the efficacy of nivolumab in combination with the anti-LAG-3 monoclinal antibody relatlimab or in combination with ipilimumab (NCT04080804).

#### 7.2.3. B7-H3

Similar to B7-H1 (CD274), B7-H3, a member of the B7 ligand family, is also overexpressed in HNSCC [113,114]. The efficacy of the anti-B7-H3 antibody enoblituzumab was evaluated in a phase I study in combination with pembrolizumab in PD-L1-naïve, relapsed, metastatic HNSCC after platinum treatment. In total, 33% (6/19) of patients achieved an overall response rate [115]. Concurrent trials are ongoing to evaluate the efficacy of enoblituzumab in combination with the retifanlimab (anti-PD-1) or tebotelimab molecule designed to target LAG-3 and PD-1 in patients with recurrent metastatic HNSCC (NCT04634825).

#### 7.2.4. NKG2A

NKG2 proteins are receptors on natural killer (NK) cells. There are seven types: A-H. These receptors can be either activating or inhibitory, depending on the dimerization partner and depending on the type of receptor. NKG2A is an inhibitory molecule, an immune checkpoint inhibitor [116]. Monalizumab (anti-NKG2 antibody) is being tested on tumor infiltrating CD8+ T lymphocytes and NK cells. When combining monalizumab and cetuximab, the data showed an overall survival of 36% in patients who have not been treated with immunotherapy and 17% in pretreated patients, with an overall survival of 44% after 12 months [117]. The cohort was expanded and durvalumab was added. This resulted in an overall response rate of 33% and a median overall survival of 15 months [118]. The study has progressed to phase III (NCT04590963).

#### 7.2.5. TLR9

TLR9 promotes tumor regression by triggering a cytotoxic T cell (CTL) response and reducing the number of MDSCs, tumor-associated macrophages (TAMs), and Tregs [119]. Dendritic cells can be activated by the synthetic CpG-ODN agonist SD-101 to secrete IFN-α, become APCs, and activate T cell anti-tumor responses. Pembrolizumab is currently being investigated in combination therapy with SD-101. This is being performed on R/M head and neck patients who do not express PD-1 [120].

#### 7.2.6. Cellular Therapy

TIL isolated from primary tumors and genetically engineered T cell receptor T cell and NK cell-therapies (chimeric antigen receptor (CAR) T cell/NK-cell therapies) have shown clinical efficacy in a subset of solid cancers.

CAR T cells/NK cells are therapies of a target antigen specific nature. A clinical phase II trial is determining the clinical response rate (CR+PR) with irradiated PD-L1 CAR-NK cells in combination with N-803 plus pembrolizumab in patients with head and neck squamous cell carcinoma and gastric/GEJ cancer (NCT04847466). N-803 (Anktiva) is a mutant IL-15-based immunostimulatory fusion protein complex (IL-15RαFc) that stimulates the proliferation and activation of NK cells and CD8+ T cells, but not regulatory T cells [121].

HPV-positive patients (with and without cervical cancer) with recurrent carcinoma were treated with autologous tumor-infiltrating lymphocytes. These were engineered to respond to viral E6 and E7 antigens. After lymphocyte-depleting conditioning, high-dose aldesleukin (interleukin-2) was administered systemically before cell infusion. Patients without cervical cancer revealed an overall survival of 19% (2/11 patients) [122].

### 7.3. ICI Combination Therapy against Mechanisms which Inhibit T Cell Functions

As mentioned in the previous chapter, indoleamine-2,3-dioxygenase (IDO) is able to attenuate T cell function. ECHO-202/KEYNOTE-037 and ECHO-204 tested epacadostat (IDO1 enzyme inhibitor) [123]. The combination with pembrolizumab (ECHO-202) revealed a disease control rate of 61% and an overall response rate of 34%. The most prevalent treatment-related adverse events were 11% nausea, 11% weight loss, and 24% fatigue. These data, suggesting promising anti-tumor activity with good tolerability, have led to plans for a phase III trial [124]. The combination with nivolumab (ECHO-204) had a similar efficacy with an ORR of 23% and a disease control rate of 61%. Unfortunately, the phase II trial was discontinued prematurely due to disappointing results in other tumor entities.

While antibody therapy against the PD-1/PD-L1 axis is highly effective in various types of cancer, some malignancies develop resistance to therapy. One mechanism by which this occurs is the upregulation of alternative immune checkpoints, such as CTLA-4. The surface molecule CTLA-4, when bound to B7, prevents interaction with the co-stimulatory CD28, leading to the inhibition of T cell proliferation and IL-2 production, thereby attenuating the immunologic response. Therefore, the combination of multiple checkpoint inhibitors is useful to improve response rates and survival. For HNSCC, promising inhibitors against co-stimulatory/inhibitor proteins have been developed for this purpose in recent years [125].

The dual blockade of PD-1/PD-L1 and CTLA-4 in HNSCC is currently being investigated in different studies. So far, a positive trend towards OS is suggested in a subset of patients whose tumors express PD-L1 with a CPS greater than or equal to 20. However, some phase III studies investigating the combination therapy of PD1/PD-L1 inhibitors, such as nivolumab (CheckMate 651) or durvalumab (EAGLE and KESTREL), combined with anti CTLA-4-mAb (e.g., ipilimumab or tremilimumab), did not reveal a statistically improvement in OS compared to the EXTREME treatment regimen or standard of care in patients with R/M HNSCC (AstraZeneca communication https://www.astrazeneca.com/media-centre/press-releases/2021/update-on-kestrel-phase-iii-trial-for-imfinzi.html, accessed on 22 September 2022 [60,78]).

Another promising candidate for checkpoint regulator combination therapy is the surface molecule LAG-3. The anti-LAG-3 antibody BMS-986016 is being investigated in a phase I/IIa dose escalation and expansion study (CA224-020), alone and in combination with nivolumab in advanced solid tumors, including an HNSCC cohort (NCT01968109). LAG-3 ensures the suppression of CD4+ and CD8+ T cell proliferation and activation and is also expressed on Tregs, where it facilitates their suppressive function [126,127]. 

## 8. Combination with Other Immunomodulators

In HNSCC, several signaling pathways are activated which, among other consequences, lead to an inhibition of the local immune response. An interruption in these signaling pathways in the tumor process is a goal of targeted therapies. Some of them are listed here. 

### 8.1. CXCR2

AZD5069 is a selective antagonist of CXC chemokine receptor 2 (CXCR2). A number of cytokines bind to this G-protein-coupled receptor, and it is overexpressed in HNSCC and appears to promote disease [128,129]. There is an active study evaluating durvalumab in combination with AZD9150 (antisense oligonucleotide against STAT3, see next paragraph) or AZD5069 in a phase Ib/II trial in patients with advanced solid tumors and relapsed metastatic squamous cell carcinoma of head and neck (NCT02499328) [130]. 

### 8.2. STAT3

AZD9150 (Danvartirsen) inhibits the signal transducer and activator of transcription 3 (STAT3), and has shown activity against lymphoma and lung cancer in preclinical studies. In addition, the inhibition of STAT3 sensitizes HNSCC to chemotherapy and radiotherapy, particularly nasopharyngeal carcinoma (NPC) [131,132].

The phase Ib/II SCORES trial for advanced solid tumors, including R/M HNSCC, showed that durvalumab in combination with AZD9150 (35 patients in this arm with 15 patients receiving prior PD-L1 treatment) achieved an objective RR of 25% in 20 CPI-naive patients, with a disease control rate (DCR) of 45% at 12 weeks and 30% patients still on treatment at 25 weeks. Overall, the combination was found to be well tolerated. These initial data are promising, and further results are expected. In the PD-L1-pretreated group, one complete response and one unconfirmed response were reported, with a DCR of 20% at 12 weeks [120]. 

### 8.3. EGFR

The epidermal growth factor (EGF) receptor, a member of the ERbB/HER family, is overexpressed in HNSCC at over 90%. The EGFR blockade is indicated in combination with chemotherapy as a first-line therapy for patients with metastatic disease. Cetuximab is a chimeric IgG1 monoclonal antibody that blocks EGF receptor activation and facilitates its internalization.

Anti-EGFR antibodies have also been shown to cross-prime NK and dendritic cells, which may additionally lead to tumor-specific cellular immunity [133]. Six months of treatment with cetuximab in combination with pembrolizumab has been very beneficial for patients considering an overall response rate of 45% (95% CI 28–62) [134].

The combination of pembrolizumab with the EGFR tyrosine kinase inhibitor afatinib resulted in a median progression-free survival of 4.1 months, an overall survival of 8.4 months, and an overall response rate of 41% in patients with platinum-refractory metastatic head and neck cancer [135]. Nivolumab in combination with cetuximab achieved an overall response rate of 22% in patients previously treated with cetuximab or immune checkpoint inhibitors. Depending on the response to prior therapy, the benefit of treatment could be calculated [136,137]. The combination of cetuximab with the PD-L1 inhibitors durvalumab or avelumab has also been shown to be effective, with confirmed immunostimulatory effects [138,139]. 

### 8.4. VEGF

A first step that tumors take for their growth is the recruitment of blood vessels. Angiogenesis is essential for HNSCC growth as well as invasion and metastasis. One of the main signaling routes for angiogenesis is the vascular endothelial growth factor (VEGF) signaling pathway [140].

Lenvatinib is a potent tyrosine kinase inhibitor [141]. When cetuximab was combined with lenvatinib, an overall response rate of 67% and a progression-free survival of 3.6 months were observed in a dose-de-escalation (I/Ib) study [142]. Pembrolizumab was combined with lenvatinib in one study (I/Ib) and showed an overall response rate of 46% and a median progression-free survival of 4.7 months in 22 patients with head and neck tumors. This combination has since been approved by the FDA for renal cell carcinoma. Additional studies for recurrent metastatic head and neck cancer have now reached a higher stage III (NCT04199104, NCT04428151) [143,144,145].

Furthermore, the efficacy of the VEGF inhibitor bevacizumab is currently being evaluated in combination with chemotherapy and atezolizumab (ICI), compared with cetuximab plus chemo in recurrent and metastatic head and neck cancer patients (NCT05063552). 

### 8.5. PDE5

Phosphodiesterase-5 (PDE5) plays a key role in regulating a variety of *cyclic* guanosine monophosphate (*cGMP*)-mediated physiological processes involving multiple regulatory mechanisms, including allosteric structural changes and post-translational modifications (such as phosphorylation). A number of pharmacological inhibitors for the treatment of a range of diseases, including erectile dysfunction and pulmonary hypertension, employ PDE5 inhibitors. There is growing evidence that PDE5 inhibitors play a role in treating several other diseases, including cancer and COVID-19 complications. It has been shown that tadalafil, a PDE5 inhibitor, augmented an immune response in HNSCC, increasing ex vivo T cell expansion to a mean 2.4-fold increase compared to a 1.1-fold increase in control patients (*p* = 0.01), reducing peripheral MDSC numbers to a mean 0.81-fold change compared to a 1.26-fold change in control patients (*p* = 0.001), and increasing general immunity as measured by delayed-type hypersensitivity response (*p* = 0.002) [146]. A phase II trial examines the combination of pembrolizumab and tadalafil for safety and efficacy in advanced head and neck cancer (NCT03993353).

### 8.6. SMO

Smoothened (SMO), as a transmembrane protein, is a key component of the Hedgehog signaling pathway, a cell–cell communication system critical for embryonic development and adult tissue homeostasis [147]. Aberrant Hedgehog signaling causes birth defects and cancer [148]. The G protein-coupled receptor SMO conducts the signals across the membrane [149]. Targeting SMO is considered as a therapeutic option in patients with head and neck cancer [148]. A phase I study is currently examining the influence of the SMO inhibitor Sonidegib in combination with pembrolizumab on the spreading of solid tumors (metastatic HNSCC) in the body (NCT04007744) [150].

### 8.7. Aurora Kinase A (AURKA)

The protein family consists of three members: Aurora A (AURKA), Aurora B (AURKB), and Aurora C (AURKC). Both AURKA and AURKB play important roles in regulating cell division during mitosis, while AURKC plays a unique physiological role in spermatogenesis. AURKA and AURKB have been found to function as oncogenes and promote tumorigenesis in various cancers, including solid tumors and hematologic malignancies [151].

Apart from the role that AURKA plays in mitosis, more and more studies suggest that AURKA, when abnormally expressed, may be an oncogene involved in tumorigenesis [152]. Gene amplification, transcriptional activation, and the inhibition of protein degradation could contribute to increased AURKA expression in cancer tissues. AURKA promotes tumorigenesis by participating in cancer cell proliferation, epithelial–mesenchymal transition (EMT), metastasis, apoptosis, and the self-renewal of cancer stem cells. Since an overexpression and gene amplification of AURKA have been found in various cancers, small-molecule AURKA kinase inhibitors are of great interest [153]. Some Aurora kinase inhibitors (AKIs) have already been used in clinical trials. The AKI alisertib has completed phase III clinical trials for patients with relapsed/refractory peripheral T cell lymphoma (NCT01482962) [154]. Moreover, there is currently an ongoing trial investigating the efficacy of alisertib in combination with pembrolizumab in treating patients with Rb-deficient head and neck squamous cell cancer (NCT04555837) [155].

### 8.8. PARP

Poly (ADP-ribose) polymerases (PARPs) are a family of related enzymes known to catalyze the transfer of ADP-ribose to target proteins. PARPs play an important role in various cellular processes, including in the modulation of chromatin structure, transcription, replication, recombination, as well as DNA repair. The role of PARP proteins in DNA repair is of particular interest because certain tumors in which homologous recombination is impaired require PARP-mediated DNA repair for survival and are sensitive to its inhibition. PARP inhibitors may also increase the sensitivity of tumors to DNA-damaging agents. Clinical trials with PARP inhibitors are investigating the utility of these approaches in cancer [156]. HNSCC is characterized as an immunosuppressive disease with aberrant DNA repair pathways, and it has been shown that HNSCC may be a good candidate for PARP inhibitor-based treatment strategies [157]. Currently, the PARP inhibitor olaparib is being investigated in combination with pembrolizumab and carboplatin as a first-line treatment of R/M HNSCC in a phase II clinical trial (NCT04643379).

### 8.9. EZH2

The enhancer of zeste homolog 2 (EZH2) is an enzymatic catalytic subunit of polycomb repressive complex 2 (PRC2) that can alter downstream target gene expression via the trimethylation of Lys-27 in histone 3 (H3K27me3). Since EZH2 regulates cell cycle progression, the dysregulation of EZH2 increases cell proliferation and prolongs cell survival, which may lead to cancer formation and progression [158]. EZH2 modulates the EMT of hypopharyngeal cancer cells in a Snail/Slug-dependent manner and is associated with more advanced T stage and poor prognosis in HNSCC [159]. A phase II clinical trial investigates tazemetostat, a EZH2 inhibitor, in combination with a fixed dose of pembrolizumab in patients with R/M HNSCC (NCT04624113).

### 8.10. PPAR-α

Peroxisome proliferator-activated receptors (PPARs) are among the three ligand-inducible transcription factors belonging to the nuclear receptor superfamily. PPARs have been found to play a vital role in regulating the expression of a variety of genes related to glucose and lipid metabolic homeostasis, adipogenesis, and inflammation. There is growing evidence for the effects of PPAR-α and PPAR-γ on carcinogenesis, which overlap in the areas of metabolism and inflammation modulation [160]. TPST-1120 is a first-in-class oral therapy that inhibits PPAR-α, a transcription factor that regulates fatty acid oxidation (FAO). TPST-1120 has demonstrated multiple modes of anti-tumor action in preclinical studies in advanced solid tumors, including an inhibition of tumor proliferation, an increase in the anti-angiogenic factor thrombospondin 1, and a reduction in T cell exhaustion [161]. A phase I study evaluates TPST-1120 as a type of monotherapy used in combination with nivolumab in subjects with advanced solid tumors, including HNSCC (NCT03829436), among others.

### 8.11. PTPN2

Tyrosine protein phosphatase non-receptor type 2 (PTPN2) is an enzyme that, in humans, is encoded by the PTPN2 gene. The encoded protein is a member of the protein tyrosine phosphatase (PTP) family. PTPs are known signaling molecules that regulate a variety of cellular processes, such as cell growth, differentiation, mitotic cycle, and oncogenic transformation [162]. The CRISPR-mediated knockdown of PTPN2 sensitized the response to anti-PD1 treatment by enhancing IFN-γ-mediated antigen presentation and increasing cytotoxic Tim-3+ CD8+ T cells [163,164]. The novel PTPN2 inhibitors ABBV-CLS-579 (NCT04417465) and ABBV-CLS-484 (NCT04777994) are currently under investigation in combination with anti-PD1 therapy in phase 1 clinical trials for locally advanced and metastatic solid tumors, including HNSCC.

### 8.12. TGF-β

The simultaneous inhibition of the PD-1/PD-L1 and TGF-β axes enhanced anti-tumor immunity [165]. M7824 (Bintrafusp alpha) is designed to simultaneously target two immunosuppressive signaling pathways (TGF-β-Trap and PD-L1). Fifty-nine patients with advanced and pretreated checkpoint inhibitor-naive HPV-associated cancers demonstrated a total clinical response rate of 35.6% (95% CI, 23.6% to 49.1%) in phase I and phase II trials (NCT02517398 and NCT03427411) [166].

### 8.13. Vaccines Based on Peptide–Protein

Peptide vaccines can be used to selectively kill tumor cells. The vaccines contain a synthetic epitope specifically expressed on tumor cells.

The vaccine ISA101 contains peptides against the E6 and E7 proteins of HPV16. In a clinical trial (phase II), ISA101, in combination with the immune checkpoint blockade, shows an overall response rate of 33% in HPV16-positive oropharyngeal carcinomas [167]. In a new study by de Sousa et al., twenty-four patients were monitored for a median of 46.5 months. The median duration of response was 11.2 months; 38% of patients with an objective response did not show any progression at 3 years. The median overall survival reached 15.3 months (95% CI, 10.6 months to 27.2 months). The 3-year overall survival rate was 12.5% (95% CI, 4.3% to 36%). The two patients with complete responses had the highest percentage of CD8+ T cells at the same time. In addition, there was significant differential gene regulation (*p* < 0.05) of 357 genes (≥1.25-fold) between responders and non-responders. An expression of the interferon pathway, immune response, and inflammatory response genes were also correlated with a better clinical response (*p* < 0.05) [168].

Furthermore, DPX-E7, a peptide-based vaccine used to treat HPV-associated head and neck, cervical, or anal cancer, is currently under investigation (NCT02865135). Moreover, a phase I study with CUE-101, an E7-pHLA-IL2-Fc fusion protein, has been shown to enhance T cell activation for the treatment of HPV16-associated cancers. Cue-101 monotherapy was used in second-line treatments, or in combination with pembrolizumab in first-line patients with PHV16+ recurrent/metastatic HNSCC (KEYNOTE-A78 and NCT03978689) [169,170].

### 8.14. Vaccines Based on Nucleic Acids

Nucleic-acid-based vaccines are based on the administration of messenger RNA, which is then transcribed by cells into a corresponding protein. Treatments with such vaccines produced a good response and durable immune responses in a clinical trial (phase I/II) [171]. Durvalumab, combined with the vaccine MEDI0457, demonstrated an overall response rate of 22.2% with three partial responses and one complete response in an open-label multicenter study (NCT03162224). Patients with incurable histologically/cytologically confirmed R/M HPV+ HNSCC who had ≥1 prior platinum-containing therapy or other approved therapy were administered by MEDI0457 along with durvalumab, until disease progression or unacceptable toxicity was achieved [172].

## 9. Common Clinically Applied ICIs of PD-1/PD-L1 Axis and Combination Therapy in Early Clinical Phases

As outlined above, ICIs targeting the PD-1/PD-L1 axis have provided a wide variety of therapeutic options in recent years. Especially with regard to tumor resistance and metastatic spread, some combinations of diverse therapeutics with ICIs achieved excellent response rates and improvements in patient survival and quality of life, enabling them to progress to higher clinical phases.

**Figure 3:**
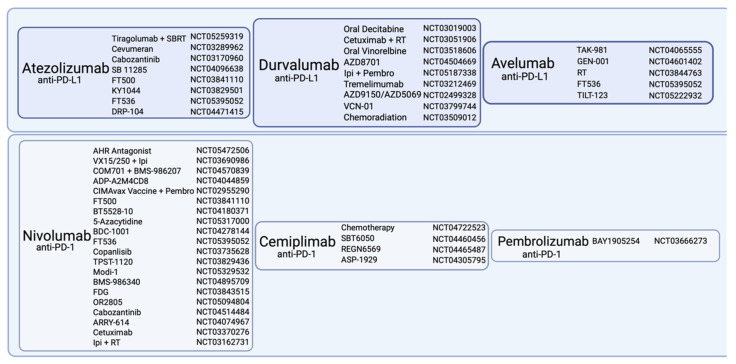
List of early-phase studies of common clinically used ICIs in combination with various therapeutics and radiation modes (created with Biorender.com).

There is now a process of refining the results which is reflected in countless studies of PD-1/PD-L1-ICI axis combination therapies which are in early phases. Figure 3 provides a comprehensive list of early-phase studies investigating novel and/or expanded combinations with ICIs, as well as the extension of combinations of radiotherapies and ICIs to many types of solid tumors.

## 10. Immunotherapy Combined with Chemoradiotherapy

Looking at unselected patients with HNSCC, response rates are relatively low compared with other tumor entities, ranging from 10 to 20% [52,173]. Therefore, better patient selection is needed, which requires the use and research of preventive biomarkers, and an enhancement of the specific anti-tumor immune reaction by other therapies may further improve the response rates of immunotherapy.

The range of possible combinations goes from specific established antibodies to small molecules to radiation therapy. The immunomodulatory effects of radiotherapy, which have recently been reported, are now leading to a growing interest in synergistic effects with immunotherapy.

An effective method of provoking the immunogenic death of tumor cells, in addition to the application of immune checkpoint inhibitors alone or with chemotherapy, is the combined treatment with radiotherapy.

This combination becomes interesting in cases where PD-L1 is upregulated in tumor cells after chemo-radiotherapy. From our data, we observe a change in the localization and expression level of PD-L1 in a number of head and neck tumor cell lines. In addition, several preclinical studies have demonstrated the upregulation of PD-L1 in tumor cells after chemo-radiotherapy (CRT) [174,175].

The combination of adjuvant durvalumab and CRT in locally advanced and unresectable NSCLC resulted in an improvement in RR and PFS, as well as in the median time to death, according to a phase III PACIFIC study [176].

In a pilot study of HPV-associated locally advanced oropharynx tumors from predominantly male patients (90%), CRT increased the number of CD8+ T effector cells, CD4+ regulatory cells, and T cells with PD1, TIM3, and LAG3 expression [177].

In addition, the combination of pembrolizumab with cisplatin-based CRT was demonstrated to be well tolerated in locally advanced HNSCC. In the study, 27 patients with predominantly HPV-positive oropharynx tumors (74%) were given one dose of pembrolizumab 4-7 days before CRT, followed by three weekly doses during CRT and five doses after the completion of CRT. Three of the patients had to discontinue treatment due to immunological side effects. However, 85% achieved the targeted cisplatin dose and 78% completed the planned doses of pembrolizumab. This trial has now been extended to additional HPV-positive and -negative cancer cohorts to confirm tolerability and provide preliminary evidence of efficacy [178].

The combination of cetuximab and RT for the radical treatment of locally advanced HNSCC affects dendritic cell maturation [133,179] and data suggest that it increases the expression of inhibitory checkpoints on TILs [180,181]. The phase III clinical trial REACH (NCT02999087) is investigating the clinically used PD-L1 antibody avelumab in combination with cetuximab–RT. This study uses data which indicate that both cetuximab and avelumab activate the antibody-dependent cellular cytotoxicity (ADCC) signaling pathway [179,182].

## 11. Checkpoint Inhibition in Combination with Viral Therapy

A number of preclinical studies suggest that oncolytic viruses can successfully suppress tumors by provoking an immunological response against tumors. It has already been shown that tumor burden can be reduced [183,184]. Resistance to checkpoint inhibitors can be overcome in combination therapy with PD-1 inhibitors [184]. KEYNOTE-137 (NCT02626000) is an ongoing phase Ib/III randomized trial which evaluates the combination of talimogene laherparepvec (T-VEC) with pembrolizumab in R/M HNSCC. T-VEC has already been approved for unresectable metastatic melanoma. It is a modified live-attenuated herpes simplex virus type 1 designed to promote an anti-tumor response by selectively replicating tumor cells and producing a granulocyte–macrophage colony-stimulating factor (GM-CSF) to stimulate systemic anti-tumor immunity [185,186].

## 12. Side Effects

The side effects of CPIs, immune-related adverse events (irAEs), play a role during treatment. These autoimmune side effects occur more frequently in patients with pre-existing autoimmune diseases.

A retrospective analysis of nivolumab side effects in advanced melanoma found irAEs in 49% of the 576 patients examined. Gastrointestinal, skin, liver, and hormonal side effects were the most common. Only 4% of patients had severe grade 3-4 irAEs. Furthermore, 24% of patients required systemic immunosuppressive treatment, and in most cases the side effects resolved on their own [187].

The side effect profile of nivolumab in HNSCC patients in the CheckMate 141 trial showed lower rates of gastrointestinal and hepatic toxicities compared to standard treatment. However, increases in skin toxicity (15.7%), endocrinopathies (7.6%), and pneumonitis (2.1%) were reported [60].

Although unpleasant for the patient, the occurrence of irAE seems to be correlated with a better therapeutic outcome in general. In the 2018 ASCO study of 114 patients with metastatic HNSCC, 59 irAEs occurred in 49 patients. ORRs in patients with irAEs were much higher (30.6% vs. 12.3%, *p* = 0.02). PFS (6.9 vs. 2.1 months; *p* = 0.0004) and mOS (12.5 vs. 6.8 months; *p* = 0.007) were also significantly better in patients with irAEs than in those without. A pooled analysis of patients suffering from metastatic melanoma showed similar results regardless of whether they received systemic immunosuppression to treat their irAEs or not [187].

## 13. Conclusions

Immunotherapy has dramatically changed the treatment of advanced and recurrent/metastatic HNSCC by improving overall survival and reducing toxicity compared to conventional treatment strategies. However, treatment responses are still limited to a reduced number of patients. Currently, great efforts are made to better understand the immunogenic processes involving both tumor cells and tumor environments that are associated with treatment responses in order to manipulate and enhance efficacy. Combinations of immune checkpoint inhibitors with monoclonal antibodies, chemo- or radiotherapies, as well as other strategies (such as oncolytic virotherapy, vaccination, or CAR-T cell therapy) are under investigation in clinical and pre-clinical studies. Further biomarkers for treatment response shall help with patient stratification in future therapies.

## Figures and Tables

**Table 1:** Clinical phase III trials for the treatment of HNSCC patients with ICI.

Treatment Setting	Trial	Description	Objective	Results
ICI–chemo	KEYNOTE 048 (NCT02358031)	Pembrolizumab monotherapy vs. pembrolizumab + platinum-based CT + 5-FU vs. cetuximab + platinum-based CT + 5-FU	Pembrolizumab as a first-line treatment of R/M HNSCC.	OS over SOC was improved with pembrolizumab alone in populations with PD-L1 CPS ≥ 20 (*p* = 0.0007) and CPS ≥ 1 (*p* = 0.0086). Pembrolizumab + CT significantly improved OS in the total population (*p* = 0.0034) [52].
Dual checkpoint blockade	CheckMate 651 (NCT02741570)	Nivolumab + ipilimumab vs. SOC (EXTREME study regimen) as first-line treatment in patients with R/M HNSCC	Combination nivolumab + ipilimumab has shown significant promise in patients with NSCLC, advanced melanoma, and advanced RCC.	Trial failed end point. OS for dual immune checkpoint blockade 13.9 months vs. 13.5 months for the EXTREME group. Higher OS for double immune blockade when CPS > 20 (17.6 months), but also n.s., ORR 34%, and DOR 32.6 months. No single nivolumab arm for comparison [53].
Dual checkpoint blockade	CheckMate 714 (NCT02823574)	Nivolumab + ipilimumab vs. nivolumab + ipilimumab placebo in R/M HNSCC	R/M HNSCC ORR, DOR, TTR.	Study aim failed: OS for nivolumab plus ipilimumab 10.0 months vs. 12.0 for nivolumab plus placebo. ORR: 13.2 for nivolumab plus ipilimumab vs. 18.3 for nivolumab plus placebo.
Dual checkpoint blockade	KESTREL (NCT02551159)	Durvalumab + tremelimumab vs. durvalumab monotherapy vs. SOC CT in treatment-naive R/M HNSCC patients	First-line treatment for R/M HNSCC targeting both PD-L1 and CTLA-4 pathways has potential for synergistic anti-tumor effects.	Results ongoing [54].
Dual checkpoint blockade	EAGLE (NCT02369874)	Durvalumab monotherapy vs. durvalumab + tremelimumab vs. SOC in R/M HNSCC with progress on platinum therapy	Second-line treatment for R/M HNSCC targeting both PD-1 and CTLA-4 pathways may induce synergistic anti-tumor effects.	Did not meet primary endpoint of improved OS [55,56].
Single ICI adjuvant	WO40242 (NCT03452137)	Atezolizumab vs. placebo for high-risk stage IV HPV- or stage III HPV+ HNSCC after definitive local therapy	To evaluate the efficacy and safety of atezolizumab as an adjuvant therapy.	Primary outcomes include independently assessed event-free survival (IRF assessed EFS) and OS.
ICI–chemo- radiation	GORTEC 2017– 01 (REACH) (NCT02999087)	Avelumab + cetuximab and RT vs. SOC in LA HNSCC	The expansion of GORTEC 2015–01, based on the hypothesis of a synergistic benefit when avelumab is combined with cetuximab + RT.	This study demonstrated an acceptable safety profile and was approved for continuation by the Data and Safety Oversight Committee [57].
ICI–radiation	JAVELIN (NCT02952586)	Avelumab + SOC CRT vs. SOC CRT in LA HNSCC patients	The combination of avelumab and CRT may synergistically activate multiple immune-mediated mechanisms and improve long-term disease control [58].	Currently recruiting.
ICI–radiation	KEYNOTE-412 (NCT03040999)	Pembrolizumab or placebo + CRT in LA HNSCC patients	CRT exhibits immunomodulatory effects; preclinical data indicate efficacy may be improved with the addition of pembrolizumab [59].	Adult patients with newly diagnosed, pathologically proven, untreated LA-HNSCC are being recruited [59].
ICI–radiation	(NCT03349710)	Nivolumab monotherapy vs. nivolumab + cisplatin in combination with RT in cisplatin ineligibility or eligibility will be assessed in LA HNSCC patients	To evaluate whether nivolumab in combination with RT is more efficient compared to cetuximab in combination with RT.	Recruitment completed. *n* = 74. AE, SAE evaluation.
Dual checkpoint blockade, ICI–radiation, adjuvant–neoadjuvant	IMSTAR-HN NCT03700905	Multicenter randomized controlled study of nivolumab alone or in combination with ipilimumab as an immunotherapy vs. standard follow-up in surgical resectable HNSCC after adjuvant therapy	The combination of anti-PD-1 and anti-CTLA-4 as maintenance therapy may improve DFS due to the anti-tumor effect of immunotherapy by enhancing the cross-presentation of tumor antigens. Primary: DFS at 3 years.	Active, not recruiting, 276 participants estimated.
ICI–chemo, ICI–radiation	NCT01810913	Docetaxel–cetuximab or the addition of an immunotherapy drug, atezolizumab, to the usual chemotherapy and radiation therapy in high-risk HNSCC	DFS, OS.	Active, recruiting, 613 patients estimated.
ICI–radiation	NCT03258554	Radiation therapy with durvalumab or cetuximab in treating patients with locoregionally advanced head and neck cancer who cannot take cisplatin	It is not clear whether radiation therapy with durvalumab is more effective than usual radiation therapy with cetuximab in treating patients with head and neck cancer (DLT, PFS, OS).	Recruitment suspended.
ICI–AB–chemo	NCT05063552	An evaluation of the application of the investigational drugs atezolizumab and/or bevacizumab with or without standard chemotherapy in the second-line treatment of advanced head and neck cancer	To investigate the progression-free survival (PFS) of patients receiving chemotherapy plus cetuximab, chemotherapy plus bevacizumab, and atezolizumab plus bevacizumab (phase II). To assess the overall survival (OS) of patients treated with chemotherapy plus cetuximab versus the superior arm from the phase II portion of the protocol (phase III).	Recruiting.
ICI–AB	NCT04199104	A trial of pembrolizumab with or without lenvatinib (E7080/MK-7902) as a first-line treatment (1 L) in a programmed cell death ligand 1 (PD-L1)-selected population with recurrent or metastatic squamous cell carcinoma of the head and neck (R/M HNSCC). (LEAP-010) (MK-7902-010) (LEAP-10)	ORR, PFS, OS.	Recruiting.

ICI: immune checkpoint inhibitor, AB: antibody, ORR: objective response rate, DOR: duration of response, SOC: standard of care, OS: overall survival, DFS: disease-free survival, PFS: progression-free survival.
